# “Give Us the Chance to Be Part of You, We Want Our Voices to Be Heard”: Assistive Technology as a Mediator of Participation in (Formal and Informal) Citizenship Activities for Persons with Disabilities Who Are Slum Dwellers in Freetown, Sierra Leone

**DOI:** 10.3390/ijerph18115547

**Published:** 2021-05-22

**Authors:** Victoria Austin, Cathy Holloway, Ignacia Ossul Vermehren, Abs Dumbuya, Giulia Barbareschi, Julian Walker

**Affiliations:** 1 Development Planning Unit & GDI Hub WHO Global Collaborating Centre for AT, University College London, London WC1E 6BT, UK; 2 UCLIC Engineering & GDI Hub WHO Global Collaborating Centre for AT, University College London, London WC1E 6BT, UK; c.holloway@ucl.ac.uk (C.H.); giulia.barbareschi.14@ucl.ac.uk (G.B.); 3 Development Planning Unit, University College London, London WC1E 6BT, UK; ignacia.ossul@ucl.ac.uk (I.O.V.); julian.walker@ucl.ac.uk (J.W.); 4 Dorothy Springer Trust, Opportunities House, 18 Race Course Road, Cline Town, Freetown, Sierra Leone; abs_d2004@yahoo.co.uk

**Keywords:** disability, assistive technology, citizenship, disability justice

## Abstract

The importance of assistive technology (AT) is gaining recognition, with the World Health Organisation (WHO) set to publish a Global Report in 2022. Yet little is understood about access for the poorest, or the potential of AT to enable this group to participate in the activities of citizenship; both formal and informal. The aim of this qualitative study was to explore AT as mediator of participation in citizenship for persons with disabilities who live in two informal settlements in Freetown, Sierra Leone (SL). The paper presents evidence from 16 participant and 5 stakeholder interviews; 5 focus groups and 4 events; combining this with the findings of a house-to-house AT survey; and two national studies—a country capacity assessment and an informal markets deep-dive. Despite citizenship activities being valued, a lack of AT was consistently reported and hindered participation. Stigma was also found to be a major barrier. AT access for the poorest must be addressed if citizenship participation for persons with disabilities is a genuine global intention and disability justice is to become a reality.

## 1. Introduction

The World Health Organisation (WHO) estimates that there are currently one billion people in the world who need access to assistive technology (AT). Yet over 90% currently do not have access to assistive products (AP)—such as wheelchairs, hearing aids, walking sticks and eyeglasses—they need, nor and the systems and services necessary to support their appropriate provision [1]. This shocking deficit is set to double by 2050, with about two billion of us likely to require AT but no anticipated reduction in lack of access. The World Health Organisation defines AT as the “the umbrella term covering the systems and services related to the delivery of assistive products and services”, which are products that “maintain or improve an individual’s functioning and independence, thereby promoting their well-being” [2], and the importance of AT provision is strongly highlighted in the Convention on the Rights of Persons with Disabilities (CRPD) [3]. AT has also been shown to be essential to achieving many of the United Nation’s Sustainable Development Goals (SDG) [4]. Without access to AT, many persons with disabilities are unable to go to school, be active in their communities, earn an income, or play a full role in their families [5]. As a recent study found, “AT can make the impossible possible for people living with a wide range of impairments, but a lack of access to basic AT …excludes individuals and reduces their ability to live full, enjoyable, and independent lives” [6].

While studies focusing on AT in terms of new technology [7]; health systems [8] and partnerships [9]; access to education and livelihoods [8,10]; and COVID-19 [11,12] are increasing, there remains very little evidence concerning the impact of AT on access to participation in activities of citizenship, and even less concerned with the very poorest persons with disabilities in the Global South. There are of course some notable exceptions, such as this informal country capacity assessment undertaken for AT provision and supply [13] and this example of the social network mediating assistive technology use in informal settlements [7]. Though much previous research has highlighted the importance of persons with disabilities being able to participate in the issues that affect their lives themselves [14,15,16,17,18,19], this work rarely considers the role of AT.

For slum dwellers who are also persons with disabilities, life can be made even more difficult by a lack of access to AT. The term ‘slum dwellers’ is used as the preference of Slum Dwellers International affiliate organisation Federation of the Urban and Rural Poor (FEDURP); study partner. It is a reclaimed word and intended to challenge the pejorative assumptions about slum dwellers. For more on ‘slum’ as a contested term see [20]. Informal settlements are characterized by non-regulated arrangements of self-built housing which often lack access to basic services, and usually in situations of precarity of both location and tenure [21]. Informal settlements constitute 36% of residential land use in Freetown, Sierra Leone [22] and in 2016 it was estimated that over 60% of the urban population of Africa live in slums. Slum dweller communities tend to be poor, and since poverty and disability are critically linked [23] AT can be even more important for independence and access to livelihoods—yet there is a surprising paucity of research about this. Often persons with disabilities find resilience to precarious situations harder to navigate [21] making this an issue of significance.

The handful of studies which have investigated AT provision in informal settlements. have tended to focus on specific groups or technologies—for instance, how blind and partially sighted people navigate in informal settlements [7]; how wheelchair users overcome physical access barriers in informal settlement with mobile phones [24]; and the impact of low-cost AT-solutions for individuals with spinal cord injuries (in this case, in Bangladesh) [25]. These studies largely focused on understanding how persons with disabilities living in informal settlements leverage AT to tackle fundamental everyday activities.

Further, only a few studies have begun to look at the barriers and facilitators which influence the ability of persons with disabilities to actively participate in political and civic life in this context, for example, [26] looked at the elements that make local politics in Ghana inaccessible to many people with disabilities. The study conducted by [27] in Cameroon and [28] in Jordan had also similar aims. However, we found a distinct lack of research to date which has attempted to identify the role that AT plays in facilitating political and civic participation amongst people with disabilities living in informal settlements, nor the implications that AT can have within the wider concept of citizenship and sense of belonging in one’s community.

Our research set out to open a dialogue about the role of AT in support of citizenship participation, both formal and informal, from the perspective of slum dwellers with disabilities. Unfortunately, we must paint a picture of lack of access to AP, and therefore also the services that sit around them. The WHO P’s framework for AT includes reference to the Person (AT user), Personnel (workforce), Products, Provision (services around the products) and Policy. This framework is embedded in the WHO tools (RATA and ATA-C) which we used [1]. Our study presents evidence from primary research with participants and stakeholders in two informal settlements in Freetown, between June 2019 and December 2020. We then used the themes from this investigation to reinterpret data from a door-to-door Rapid Assistive Technology Survey (rATA) and two national studies focused on AT provision and capacity and present the combined findings here. The rATA survey is part of the newly launched Assistive Technology Assessment (ATA) toolkit [29]. We acknowledge that some of the data gathered through WHO tools, especially the rATA survey, focuses more specifically on Assistive Products than the wider services which make up good AT provision. This reflects the intention of rATA (designed to be used as efficiently as possible to inform provision) [30] and to some degree also the focus of participants, who find the absence of such AP a very pressing and tangible reality. It is a reflection on the current state of global AT provision that a comprehensive understanding of the services required to enable inclusion of AT users in society is not well understood by those who do not have access to the basic AP they need. Nor is AT provision well understood by other actors who are responsible for the provision of such devices. It is precisely for this reason we have sought to investigate qualitative and quantitative data which addresses both aspects insofar as is possible, given by the absence of AT provision evidenced here. Much of what we present is shown in relief; highlighting the spaces where AT intervention (both products and services) should be. We feel this is a vital and urgent contribution to the debate.

In this paper we explore the importance of participation in the activities of citizenship; explore how AT access mediates that participation and what other barriers are present; and finally consider the availability of AT for this group. Through our analysis, we offer an often-unheard perspectives; raising the importance of AT as necessary for participation in citizenship activities for exactly those populations who have the least access yet the greatest need to make change. We also offer policy recommendations and potential avenues for future research in this important yet under-researched area.

## 2. Context

“Disability is complex, dynamic, multi-dimensional and contested” [31] and persons with disabilities make up 15% of the global population (ibid.,). In Sierra Leone (SL), Organisations of People with Disabilities have fought—and largely won—significant legal and political rights, including ratification of the CRPD. Disability identity in SL has been shaped, in part, through the civil war (1991–2002) during which disablement was used as a weapon of war increasing the numbers of people with certain types of impairments, particularly limb amputations. The Ebola epidemic (2014–2016), and the subsequent indirect health and economic crises added further inequality [32], both within populations of persons with disabilities and between populations of persons with and without disabilities as the impact and exclusion from ‘build-back’ efforts varied. In Sierra Leone, political participation registered as the top demand in the ‘Persons with Disabilities Manifesto’ at the election in 2018:

“We, the disability community of SL, call on political parties to invest in… the inclusion and participation of PWDs in the political process”

[33]

This priority recognises that exclusion from participation in citizenship activities is one of the worst consequences of inequality because it removes the possibility for people to make their own claims for justice through collective action which might tip the power balance in their favour. There is by now a relatively well-understood ‘perverse confluence’ of differing approaches to comprehending citizenship [34].

“’Citizen’ and ‘citizenship’ are powerful words. They speak of respect, rights and dignity…[with] no pejorative uses” [35] …yet, although the “idea of citizenship is nearly universal…what it means and how it is experienced, is not”

(ibid., p. 1)

Participation in collective action practices, whether campaigning directly for formal change [36,37] or supporting more informal approaches to citizenship, [38,39,40] is a well-understood demand of the disability movement globally [41] and a prerequisite for freedom [14]. Despite this, there is very little substantive work that looks specifically at the experience of persons with disabilities who live in informal settlements in relation to citizenship and we found no extant literature that considers the role of AT in this context. We seek to begin this conversation. 

### Ethics (Context)

This study was approved by the UCL Research Ethics Committee in 2018, as part of the wider AT2030 project. The ethical approval required participants to be adults and to ensure that appropriate action was taken to consider disability and vulnerability issues appropriately. For Data A and B, community leaders and residents were invited to participate on the basis of their involvement and interest in issues of disability and their willingness to participate in the research. The settlements themselves were chosen with local partners ensuring there was a long-term relationship with a community organisation that could be maintained.

## 3. Materials and Methods 

### 3.1. Setting

#### 3.1.1. Study Location

The study site for this research was Freetown, Sierra Leone, a city which is home to just over a million of the just over 7 m population of the country identified in the 2015 Census [42]. This location was chosen as there was already an active engagement in place with the Sierra Leone Urban Research Center (SLURC) which enabled the work to be carried out within a long and robust partnership which has been designed to build capacity and participation over many years. SLURC facilitated access to the settlements through the Federation of the Urban and Rural Poor (FEDURP); a sub-set of Slum Dwellers International (the organisation representing slum dwellers globally). The data collection was carried out in two of the informal settlements in Freetown. Dworzark is known as Freetown’s large settlement, sprawling up the steep hillside overlooking the city, and comprising an estimated 16,500 people in approximately 5000 households; informal markets, hostels, religious and community centers, bars and cafés are found further into the settlement with a formal health center sitting at the entrance. We worked in an area rising up the hillside called Brazil. Thompson Bay, comprising 6000 residents, is a much smaller coastal settlement, slightly further from the center, which spreads out into the estuary as new occupants arrive and build in increasingly precarious conditions. Both are unplanned and comprise self-built, informal structures without access to running water or regularised sanitation. 

These particular settlements were chosen in consultation with FEDURP and SLURC for relevance—e.g., persons with disabilities were present in the communities—and for the communities’ own interest in building their demographic data around disability which could be used as an advocacy tool by FEDURP as part of their wider commitment to advocacy though ‘information as power’. This participation in enumeration is part of their model of building collective agency and evidence to support their claims.

#### 3.1.2. The Policy Context in SL

Sierra Leone signed the UN CRPD on the first day allowable (30 March 2007) and followed with the enactment of the Disability Act in 2011. Regular reports on CRPD progress by the Government of Sierra Leone show significant action on disability rights and the addition of a standalone pillar in the Medium-Term Development Plan [43] has also shaped an increasingly positive disability inclusive policy landscape. However, local organisation so persons with disabilities often highlight the need to ensure laws are implemented in practice, which can run at a slower timetable. The major legislation and policy statements to date (2021) do not include explicit mention of AT provision, though this is de facto necessary to meet other commitments around disability inclusion (for instance, inclusive education). The Development Plan, 2019–2023, Ref. [43] does mention assistive devices, and the Transform Freetown Strategy (2019–2022) [44] also refers to creating an ‘enabling environment for persons with disabilities’, which could also be read as such.

Informality is sometimes presented as a binary concept, but this has widely been challenged [45] and it is often more ‘blurry’ than straightforward in reality [46] with residential and economic informality interacting in multifaceted ways. While informality is often handed pejorative association in Freetown, city and national leaders (to a greater or lesser extent) acknowledge the settlements, which are between 27–61 in number depending on definition, scattered along the coast and hillside [47]. The idea that informal practices operate in complete isolation from the State is also perhaps unrealistic as [47] found, although the implementation of formal regulation and policy in the Freetown settlements was as inconsistently evidenced (ibid.).

### 3.2. Study Design 

This study is part of a research programme (AT2030.org accessed on 2 February 2021) aimed at finding out ‘what works’ to get AT to the people that need it around the world. To build a nuanced and comprehensive understanding of how people with disabilities living in informal settlements in Sierra Leone are able (or unable) to access AT, and how this impacts their civic participation, we developed the study which aimed to address the following research questions:

- Is participation in the (formal and informal) activities of citizenship valued by persons with disabilities who slum-dwellers in Freetown, Sierra Leone?
- What level of access to AT does this group have?
- How does AT, or lack thereof, mediate (formal and informal) citizenship participation?
- What recommendations can this offer to the formulation of policy and practice?

These complex and multifaceted questions could only be answered by investigations carried out with different stakeholders and using a mixed methods approach. To this end, we combined both qualitative and quantitative datasets collected through the following studies:

**STUDY A** represents the data of a Rapid Assistive Technology Assessment (RATA) house to house survey which was collected (by co-authors I.O., J.W. and SLURC) in Dwozark and Thompson Bay settlements for the AT2030 project in September 2019. This survey data identified participants for Study B.

**STUDY B forms** the major dataset for this paper, representing data collected through interviews; focus group observations; and events held with slum-dwellers with disabilities, collected by first author V.A. (and co-author A.D. on events) in November/December 2019 and December 2020, and was supported by co-authors I.O. and J.W. 

**Figure 1:**
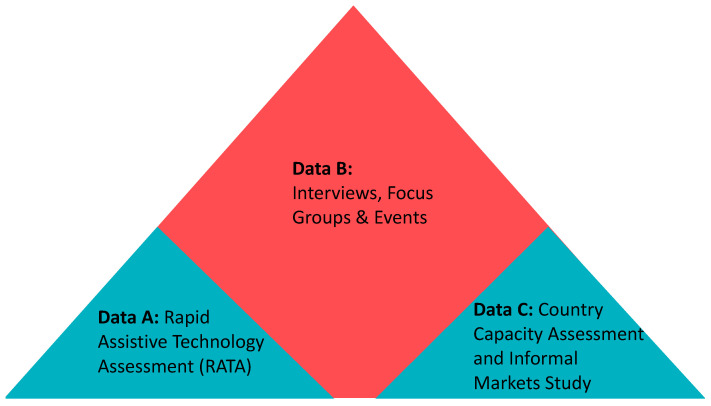
The themes from data B were used to reinterpret data A and C.

**STUDY C** represents data from two national surveys on country capacity and provision (formal and informal) of AT in Sierra Leone and was conducted by the Clinton Health Access Initiative (CHAI) and co-author JW respectively. Figure 1 shows how the different data sets have been used in combination.

Methodological details for each study and approaches for data analysis are provided in the following sections. Practically, ethically and politically this research was designed to elevate the voices of persons with disabilities who are slum-dwellers themselves—through letting the data drive the argument and in so doing seeking social justice through pedagogy [48]. With this goal in mind, we used an inductive thematic analysis approach [49] to lead the interpretation of results and grounded the conceptualization of the themes in the stories, desires and frustrations of slum dwellers with disabilities who took part in Study B. 

To strengthen and contextualize findings, we used the themes which emerged from our study with slum dwellers themselves (B), to reinvestigate data from the two other datasets (A and C). This enabled the thematic codes to be tested and nuanced and additional evidence to be added, particularly in terms of the level and type of access to AT among persons with disabilities who live in informal settlements. By combining our initial findings with quantitative data from the rATA survey and qualitative data from the semi-structured interview findings of the Capacity Assessment and informal market study (to consider how informal provision in the country as a whole influenced quality and access) we were able to build up a strong picture of AT access and its impacts for slum dwellers in Freetown.

### 3.3. Research Team and Reflexivity 

The research team for this work was led by V.A., a female, post-graduate trained social researcher and GDI Hub co-founder. V.A. lives with two mental health conditions and is British. V.A. was supported in the research by A.D., a male, post-graduate trained researcher and NGO CEO, who lives with several impairments and is Sierra Leonean. I.O., G.B., C.H., are also female, and J.W. male; all are post-graduate trained expert researchers on qualitative and quantitative studies based at UCL. Data C was supported by the Clinton Health Access Initiative, an expert organisation in AT based in SL. 

All research was funded through AT2030 and supported by SLURC. Relationships between UCL and SLURC focused on AT have been strong since the start of AT2030 in 2018, and this study was supported by a scoping visit in March 2018. 

All researchers had pre-existing expertise in working with disability and AT, and all but G.B. and C.H. have previous experience of working in SL. 

Participants were not personally known to the researcher team prior to the research commencement, though some professional contact had been established on the scoping visit with stakeholders who later participated in the study. 

### 3.4. Participant Selection and Data Collection 

#### 3.4.1. Study A: Rapid Assistive Technology Assessment Survey

The rATA survey was undertaken in September 2019 in the informal settlements city of Freetown, which has been selected as part of the broader AT2030 research project. The rATA is designed to primarily focus on data collection around access to AP. Within these communities, a total of 2076 individuals answered the survey (84% of the total addressed). Just over 3% of individuals declined to provide consent (79), and consent was not sought where no adults were present (306 households). The informal settlements surveyed are areas of Dwozark (*n* = 1207) and Thompson Bay (*n* = 1254). The strategy used was based on a ‘census’: everyone in the geographical area identified was interviewed. The specific geographical areas chosen within each settlement were selected by FEDURP. 

The data was collected and stored using KoBoToolbox [50], a suite of tools for data collection and analysis for use especially within challenging environments. Data collection was on smartphones. The rATA survey was overseen by The Bartlett Development Planning Unit, University College London (by co-author, I.O.) in partnership with the Sierra Leone Urban Research Centre, with a team of 12 enumerators drawn from the Federation of the Urban and Rural Poor. The enumerators participated in a three-day training conducted by DPU-UCL (co-author I.O.) and SLURC that was evaluated by the Nossal Institute for Global Health for the WHO. The UCL/SLURC team also analysed the findings (I.O., J.W., SLRUC) with the support of Leonard Cheshire (who also participated in the early planning and training). The findings were shared through workshops with the enumerator team. 

This was a pilot trial of the WHO rATA tool, a standardized survey protocol intended for global use, which is now finalized, digitized, translated into other languages, and available through the WHO for other projects to use [51]. 

#### 3.4.2. Study B: Direct, Detailed Inputs from Participants and Stakeholders

##### Semi Structured Interviews with Participants

This study included semi-structured interviews with persons with disabilities who were also slum dwellers (‘participants’); semi-structured interviews with stakeholders; observation of focus group discussions attended by a slightly wider group of AT users who were community members brought together for the research; and events held in the settlements for the UN ‘Disability Day’ in 2019 and 2020. A trained qualitative interviewer and AT expert (V.A.) conducted the interviews focusing on citizenship, participation, disability, and AT with the participants who were adults with impairments living in the two settlements (*n* = 16, 8 per settlement; 38% female). Interviews were held in or near the home of the interviewer, without observers and were attended by the researcher (V.A.) and two members of the FEDURP team to transcribe and translate. Interviews lasted 20–60 min. The participants had a range of impairments (including visual impairments, long-term and chronic conditions, mobility impairments, limb loss, and albinism) and were identified through the RATA as willing to participate in further discussions. The sample also represented a range of ages (from 17–70) with 6 female, 10 male participants. 

##### Interview Guide for Participant Interviews

Initially ‘citizenship’ was not a well-understood term in our test interview, so we informally workshopped translation and the use of the term with SLURC academics, community members, data collectors and seasoned FEDURP staff, highlighting the importance of test and trail of translation, especially on issues around disability. We moved to the text in the Topic Guide, shown in Appendix A, adding the qualifier “what does it mean to be a Sierra Leonean?” to the first question.

##### Semi-Structured Interviews with Stakeholders

The stakeholders were identified for interview (*n* = 5) by the first author (VA) through snowball sample. They were chosen as significant actors after discussion with the participants and SLURC. Of the stakeholder interviews, only one was female (20% female). Interviews were conducted without others present and in English with audio files and field notes taken (by V.A.) and transcription provided (by V.A.). Interviews lasted between 20–60 min and were face to face. There were no refusals to participate as participation had already been agreed through the rATA.

##### Focus Group Discussions

FGD were organized by SLURC and UCL for the AT2030 and observed by the first author between November–December 2019). Field notes were taken by the researcher (V.A.) and meeting notes were taken by FEDURP/SLURC and later used for reference. Translation was ‘live’ by the SLURC/FEDURP teams.

##### Events

A total of four ‘events’ were also held for this study, one in each settlement, around International Day of Persons with Disabilities in 2019 and 2020 where participants were invited, along with press and stakeholders, to celebrate disability. These events were planned and attended by SLURC with the authors (V.A., A.D.) in 2019, and by SLURC and A.D. in 2020. Field notes were taken by the researcher (V.A.) and meeting notes were taken by FEDURP/SLURC and later used for reference. Translation was ‘live’ by the SLURC/FEDURP teams.

##### Transcription, Translation and Consent

Translation and transcription for all data (excepting stakeholder discussions which were in English) was necessary from Krio to English. For participants, English to Krio translation was also provided (including for consent), and for research purposes, audio files were recorded using a mobile device, and later translated into a written text English file verbatim. This was not shared back with participants who (with two exceptions) did not read and write in English. This work was paid and undertaken by FEDURP members who were trained to do so by the UCL team (JW, IO, VA).

Interviews were once only and were led in English and with questions translated verbally into and from Kiro (with two exceptions where participants spoke and studied in English already). Translators were present at the interview so that follow-up questions could be asked, and field notes were also taken by the researcher to supplement the transcripts. Consent was given in writing (for AT2030) and a verbal reminder was provided at the start of the discussion.

#### 3.4.3. Study C: Country Capacity Assessments

An AT Country Capacity Assessment (CCA) was undertaken using an adapted version of a preliminary WHO tool, and an Informal Markets Study was carried out using a qualitative approach. Together these investigations make up Data C and provide more in-depth analysis of how AT is provided and obtained in SL.

##### Formal Country Capacity Assessment

The CCA tool (called ATA-C by WHO) is now available through the WHO toolkit for others to use [29]. The CCA study was commissioned (by V.A.) and undertaken by trained research practitioners at the Clinton Health Access Initiative (CHAI) SL team, in service of the GoSL Ministry of Health in the latter part of 2019. The CCA surveyed stakeholders and interviewed the participants listed in Appendix B to understand policy and practice: —what systems exist to support AP provision, which are working well, and which need strengthening for the provision of AT within the country? The data of the CCA remains the property of the government and has not been published, but for the purposes of this paper, the MoH SL released summary data to the research team.

##### Informal Markets Study

An Informal Markets Study was also commissioned (by V.A.) to investigate the capacity and provision of AT from the informal (unregulated) sector (led by JW) for which data was collected in November 2019. This study also informed the WHO ATA-C tool development and is summarized in a working paper [16] with a forthcoming journal article pending. Summary data from this study was reinterpreted in combination with the other datasets in the Citizenship study to augment the findings for this paper.

### 3.5. Data Analysis and Reporting

Taking an inductive approach, thematic analysis [49] was used to investigate the interview data with slum dweller participants. Nvivo (2020 version) was used for data management and analysis by V.A. Initial coding resulted in 27 codes. These were iterated and grouped through consultation with CH into five themes: Status in the Community; Sense of Belonging/shame; Expectations of Citizenship; Active Citizenship Practices; and (lack of) Collective Participation. These themes were used as the basis for analyzing the remaining data in Data B (focus group and observations of events) as well as Data A (RATA) and Data C (Formal County Capacity Assessment and Informal Markets Study). This allowed the themes of the investigation into the other datasets to be established by the lived experience of the individuals living with impairments in the informal settlements.

The themes continued to iterate during the analysis of Data A and Data C (by VA) as additional meaning and context were identified for each of the five themes, which were tested with evidence from each dataset. Through consultation with the wider authorship, a sixth theme emerged (AT access and Provision) and a slight readjustment of the initial 27 codes was evolved; combining and adding some new to make a total of 28. The resulting coding pattern is set out in Appendix C. It should be noted this coding framework is not intended for direct application onto other studies as this approach was designed to be context-led, letting the voices of the slum-dwellers set the direction, without extant expectations or pre-supposed coding patterns to ensure the experience of participants drove our results.

A number of strategies were implemented to ensure the rigor of the qualitative research. These included engaging a diverse and experiences research team; following clear protocols; robust data management; transparency of approach and clarity of data presentation; and reaching data saturation for findings. A core part of this was ensuring individual and collective positionality was acknowledged, discussed when relevant and reflected upon regularly in team meetings. As the data analysis progressed, these positionality differences were explored to ensure as nuanced a message as possible in this paper. Due to the global pandemic, it was unfortunately not possible to hold workshops with the participants to confirm the coding themes or share results. The themes inform the reporting of the results, set out below, which are illustrated wherever possible using direct quotes from the participants (usually translated from Krio).

## 4. Results

### 4.1. Thematic Framework 

**Figure 2:**
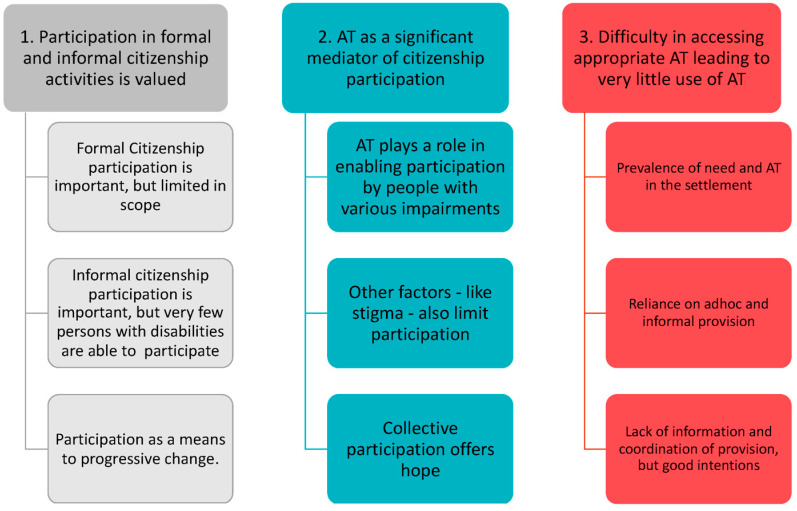
Thematic Framework of key themes and sub-themes.

The results below combine the relevant findings from Datasets A, B and C and are presented against the resulting thematic framework (see Figure 2) developed during the analysis.

The results below, are organized according to these themes, with the participants’ (from Data B) voices foregrounded, wherever possible to illustrate the argument.

### 4.2. Participatoin in Formal and Informal Citizenship Activities Is Valued by Persons with Disabilities Who Live in the Settlements

Initially, we sought to understand citizenship in context, particularly the value placed on citizenship by this group and how it operates in practice.

#### 4.2.1. Formal Citizenship Is an Important ‘Right’ but Limited in Scope in Practice

Formal citizenship participation was engaged in by all participants in data B. This formal activity operated largely in relation to activities of the State and it was important to the participants in this study that they had the right to participate. There was a particularly strong association with being proud of being from Sierra Leone. This was a typical response:

“I was born here and raised here, had my 8 children here and they attended school in SL, I am so happy to be a Sierra Leonean.”

(Woman, D-03)

Voting was the most frequent activity mentioned in relation to formal citizenship, seconded by paying taxes, and having a national ID card. Voting was felt particularly important as a ‘right’ for persons with disabilities and almost everyone went to—sometimes considerable—effort to vote in elections. For instance:

“I vote every election, I have to exercise my right to vote because it is my right.”

(Woman, D-03)

Help with moving around the inaccessible settlement is necessary to enable voting, because few participants have AT. This is offered to residents with disabilities to enable them to get to ballot boxes and cast their votes—this was mentioned often because it was partisan; offered by a political party in favor of particular candidates at election time. Often the lack of help at other times was raised as problematic and a stark comparison; several participants noted a sense of ‘disconnection’ from real change after their vote has been cast:

“…at the end of the day the election comes and they promise and then nothing is done. During the election they drive me and then I never see them again.”

(Woman, D-04)

Political participation, although universal, was largely limited to voting (where this help was available). When a young woman asked to be part of the team staffing the ballot boxes, she was told she wasn’t allowed, because of her impairment:

“Disabled people are not allowed to do things, I was told I wasn’t allowed to staff a ballot box… and I was not too ok with that, because I wanted to do it.”

(Woman, TB-01)

There was also a sense of what might be termed ‘patriotic civic participation’ which was mentioned a number of times, relating to the obligations of citizenship for to all Sierra Leoneans, including persons with disabilities, to work for peace and environmental security, given the context and recent history:

“…we are expected to keep our environment clean, to pay taxes and to …make the land become peaceful.”

(Man, D-05)

Some of these obligations—like the monthly cleaning day to prevent future landslides after a devastating one in the city—are in fact legal requirements of citizenship, enforced by the Army and Police force; even within the unregulated settlement communities. But even if the Army and police are not present it is expected (by the community and leaders) that everyone will help. Some persons with disabilities who were unable to clean themselves (often due to impairment and lack of AT) asked others to clean on their behalf to maintain their respect and status.

“I have a big position in the Scouts…so I use scouting procedure and the community people…know me, that I am…helping the community…. [for example] every cleaning [day] I organize my boys to clear.”

(Man, TB-08)

This participation was important to community recognition and status, as well as being the right thing to do.

The other way in which formal citizenship was understood was in terms of the perceptions of what the state should provide for citizens, and participants felt this was far-reaching:

“…the government should provide for our necessities because we are citizens of Sierra Leone.”

(Man, TB-07)

Often this included, but was not limited to, AT:

“[As a citizen] I should receive my education, glasses for my eyes and cream for my skin [for albinism].”

(Woman, D-02)

In common with other, non-disabled community members, it was also expected that the State should help citizens with their basic needs of life—to upgrade their living conditions and find ways to make ends meet. Participants wanted to engage with these claims:

“We are expecting good schools, good toilets, good bridges, and a good road for the community benefit…”

(Man, TB-03)

These expectations of what formal citizenship arrangements should provide for citizens with disabilities was often in reference to promises in policy and law. For example, disabled people have legal provision for free education and health services, but they were not often received, for example, a man talked about his right to free healthcare:

“…in the Disability Act…. but it doesn’t happen. So, when you go to medical (people) you have to pay. They request you to pay…. We are not getting some of the facilities we are expecting as citizens of SL.”

(Man, Dworzark)

Many of the participants talked passionately about access to education, especially for girls. Persons with disabilities often did not receive this; often they couldn’t attend school or college because of the additional costs, such as AT or books, or they were not able to access education even when fees were covered. Other times it was simply denied due to discrimination.

The reality for slum dwellers in our study was that that engagement with ‘the State’ was very limited in general, with a very strong necessary reliance on community activity and community provisioning; such as a public toilet installation, paid for by the community savings scheme in Dworzark. It is then important to understand how these informal systems of citizenship operate within the community to understand the full picture.

#### 4.2.2. Informal Citizenship Is Valued but Few Persons with Disabilities Participate

In Dworzark and Thompson Bay, community leaders include a ‘Chair’ and ‘Chair Lady’, religious and Tribal leaders (who were collectively referred to in the settlement as ‘the stakeholders’)—their role is to organize committees and lead decision making. Alongside them, the Federation of the Urban and Rural Poor (FEDURP) organize the community along the lines of other Slum Dwellers International (SDI) affiliate organisations [52] and we observed activity loosely in the categories Tomlinson (ibid,.) sets out: community organisation and capacity building; precedent-setting; knowledge exchange; partnerships and advocacy, led by FEDURP. We considered engagement with any of these as participation in informal citizenship.

Participants in our study understood that joining in informal citizenship activities—particularly community meetings and (community development) ‘projects’—was important for making a change toward the type of futures they wanted to see. When asked how they would advise someone in a similar situation to make changes, one participant said:

“I’d advise them [to] abide by the law, involve yourself in the community activities and clean.”

(Woman, TB-01)

The importance of community participation was recognized regardless of age, gender, and impairment, but sometimes these factors did determine who participated in community activities (though it is worth noting this gender divide did not apply equally to the FEDURP meetings):

“The community meeting is only for men and also stakeholders in the community.”

(Woman, TB-05)

All participants interviewed saw the value in this informal governance and supporting it, even if they did not attend meetings themselves. One man summarized this well:

“It is also important [to feeling a part of the community] to give financially, physically and morally to the development of the community.”

(Man, TB-07)

Despite this, only a few participants had ever attended community meetings and there was no evidence of disability or disability issues being raised as part of these processes (prior to AT2030). The common was response was:

“We do not have the opportunity to discuss our issues as disabled people in these meetings…they only consider the non-disabled.”

(Man, TB-02)

The organized community was mentioned by many as the most likely source of recourse to justice and support:

“Firstly, we can go to the Chief because he is the head of the community…. we should go ‘to the big one’ [Chief]…When we want to discuss these issues, we should have unity among us [persons with disabilities] so that we will later channel these issues to the stakeholder in the community, call them into community meeting, but there is no unity.”

(Man, D-01)

This lack of unity related not to disunity among persons with disabilities in the community, but rather a lack of discussion at all. There were no instances recorded of persons with disabilities meeting together or discussing issues within either settlement prior to the AT2030 project. This was for mixed reasons but often being able to get to meetings without AT, and what might be loosely termed ‘shame’ or being less valued in the community because of disability identity, featured strongly:

“I think that the reason we are not meeting together is so as not to show ourselves to the community...most of these people believe that disabled people always cause trouble...that is their knowledge.”

(Man, TB-08)

However, when asked, all participants, said they would like to meet together as a group of persons with disabilities, and could see value in this.

#### 4.2.3. Participation as a Means to Progressive Change

Collective participation was mentioned by most of the participants as desirable:

“I think that we should come together to sensitize ourselves on what we should know as disableds [sic] having one common goal. Our expectations should not be always high, but rather to advocate for support … with some AT like, wheelchair, crutches and other supporting equipment as disables, and to see how we can better our lives with this assistance.”

(Man, TB-06)

The idea of this collective participation provoked significant emotion and animation in some as many participants they did not frequently have the chance to engage with peers, or even leave the house very often and there was an emancipatory aspect about the idea of collective action to them.

“It will be good for disabled people to organize and come together and form a group because in that group you will be say the challenges you are going through, some of the struggles, be able to explain to others and other are able to proffer a solution to those challenges. By those discussions also you will be able to inform exciting opportunities that are available elsewhere.”

(Man, D-05)

Participation was not reified for its own sake—the lives of slum dwellers who are also persons with disabilities, juggling the basic needs of life and making ends meet, are hard, and time is a resource. But participation was felt to be an important ‘right’ hard won for persons with disabilities and was linked to the idea of progress, change or justice. It was suggested that if disabled people could be part of that change, they could gain respect too:

“Well if we come together…the first thing we… need is water…so if we come together as one, we work with the stakeholder in the community, we bring to other people, we bring everything together and move for water, pipes and material to come here... and make the point. As soon as water is available in the community that is the number one way [to gain respect] because the number one thing that the community does want is done by the disabled (sic).”

(Man, TB-08)

Participants largely framed participation in citizenship as an activity or process towards a future that is aspired to, no matter whether engaged in formally or informally, nor whether that change was internal (in the community) or external (by the state). Next, we turn to what mediated access to participation.

### 4.3. Mediators of Participation in Citizenship Activities 

#### 4.3.1. AT as Mediator of Participation

Many disabled participants individually, and at the observed focus groups collectively, talked about their desire to participate more, but noted the inability to because of a lack of AT.

“[Community membership] is not the same [for disabled people], because like for instance, I am having difficulty to walk, so if they call for any meeting that has to do with development, I cannot be able to attend or participate in that meeting, except I send my daughter to attend, because I am having mobility issue, so those that are the non-disabled are the ones that can be involved in the process.”

(Man, TB-06)

Some of the most striking were the older participants who wanted to make change. This participant is unable to leave her home as has no walking stick, now but she shared:

“I am not able to walk (unaided) now... …but if I were younger, I would be making bold steps, I would mobilize people.”

(Woman, D-04)

Without AT, many persons with disabilities were left unable to leave their homes and move around the settlement, or beyond, at all. This was of course made worse by the physical inaccessibility of the unplanned settlement:

“I have to stay home most of the time…you can’t use the public toilets; you can’t walk around, and no space is easy.”

(Woman, TB-01)

When asked why they did not attend meetings participants often gave the same, or a very similar, answer which related to the combined barriers of an inaccessible environment and a lack of AT:

“[Interviewer: why don’t you go to community meetings, where you say decisions are made?]. Because of physical barriers and challenges.”

(Woman, D-07)

Community leaders and stakeholders were in consensus agreement when asked why participation by persons with disabilities was lower. They said that they did not deliberately exclude persons with disabilities and wanted to work together more:

“We don’t find it difficult to accept disabled people. Whether you are disabled or not, in slum community or informal settlements you are faced with 90% the same issues. Except … there are some issues that disabled people face that able people don’t face.”

(Man, stakeholder participant)

Yet because disabled participants could not often physically get to meetings this did render them excluded, without AT or another aide. This meant community membership felt different for some:

“It is not the same [for persons with disabilities], because like, for instance, I am having difficulty to walk, so if they call for any meeting that has to do with development, I cannot be able to attend or participate so those that are the non-disabled are the ones that can be involved in the process.”

(Man, TB-06)

Often times this exclusion resulted in feelings of not being heard:

“I can tell the people with no disability that they should be listening to us as disabled, because as humans we know the starting of our lives, but we don’t know our end, there is a possibility that one day the able might also become disabled, so I say give us the chance to be part of you, and for our voices to be heard.”

(Man, TB-07)

A lack of AT was not the only reason persons with disabilities did not or were not able to participate; as the above intimates, the most referenced other reason was attitudinal barriers.

#### 4.3.2. Stigma and Discrimination Also Limited Participation

It was common for participants, for whom the lack of AT was normalized and even expected, to raise other issues relating to why they didn’t participate in citizenship activities. These often related to disability identity being largely perceived as negative as highlighted in this interview:

“In this community, the non-disabled are many and the disabled we are few and they are not seeing us as useful people. We are considered ‘less’ in this community.”

(Man, TB-06)

This was often directly experienced as stigma:

“So, you can go around [to meetings, etc.] but you choose not to because of the stigmatizations you get from people.”

(Man, D-01)

The lack of a collective voice for disabled people rendered them individually positioned against longstanding cultural assumptions and negative beliefs about disability which it was hard to tackle alone, especially when the value of persons with disabilities was considered less than that of persons without disabilities. Mockery and name-calling were also common:

“There are children who usually call me ‘cut hand’.

(Man, D-08)

The theme of disability identity, framed around usefulness and status, arose often. Participants talked about others devaluing their lives—and their deaths—because of impairment, which was deeply affecting, this wasn’t the only, but was the starkest, example:

“[When I had to have my leg amputation] at the Government hospital they gossiped, they said, “this girl may die, and that’s ok”. I was so depressed and sad and I couldn’t keep myself calm…. the non-disabled people should stop mocking the disabled people in this community because of their condition.”

(Woman, TB-1)

Sometimes indirect discrimination (or unintentional barriers) also restricted access to formal or informal citizenship activities. For instance, extra costs associated with disability which were not taken into account (e.g., the need for extra help beyond election time). Persons with disabilities also reported that they were simply unable to pay for items needed for participation (for example books) as they had much less ability to earn a livelihood than persons without a disability. In other cases, the ‘cold water’ (bribes) necessary to get access to oversubscribed services were mentioned. The lower status of persons with disabilities in society didn’t afford them the ‘sababu ‘(connections) necessary to get to the front of the line.

“We are not equal in terms of rights, because being disabled, sometimes people take advantage of us...when you have a matter in the police—someone reported you or takes something to the police station—your rights or voice as a disabled person cannot be heard.”

(Man, D-06)

While the lower status and stigma were reported many times, they were not always present. Where the participant was a community elder, then there was evidence of community help and respect:

“I find it very difficult for me to have something to eat, but with all the support of the community I am ok because they help me out...the community people do respect me as an elder person in the community.”

(Man, TB-04)

This stigma around disability identity was found inside and outside the settlement as we analyzed across data sets. But there was a particular isolation for slum dwellers who were also persons with disabilities, as this group was largely disconnected from the (relatively strong) disability movement in Sierra Leone. The main organisation of persons with disabilities, Sierra Leone Union on Disability Issues (SLUDI) and organisations of the urban poor did not work closely together. One participant has tried to encourage this:

“Their (SLUDI) offices are very far away… the last meeting I went to [in 2014] I told them that I wanted to be part of their organisations. They took my name, but they never called me... They never came here but if they did, they could sensitize the community and explain the usefulness of disabled people. So, I’m sure that if SLUDI start coming here and do some sensitization, the community will see that all the people that are part of SLUDI are (useful) disabled people.”

(Man, TB-08)

#### 4.3.3. A Desire for Collective Participation to Drive Change

When participants began to come together for AT2030, the focus group discussion observation noted comments from participants regarding a rejection of stigma issues. They wanted to invite SLUDI representatives to the community for Disability Day 2019; this small shift in the aspiration of having a collective voice for progressive change on disability, which was connected to the national disability movement, was observed as significant; representing a different picture from that reported at the start of the research (where there were zero incidences of collective participation on disability issues.) in even a few short months. This indicates the desire was beginning to be acted upon, though gives no assurance of a trend.

Stakeholder interviews, participant interviews and FGD observations found that prior to June 2019, there was one instance of a project led by stakeholders which had started to consider disability in earnest. One NGO stakeholder interviewed commented:

“...awareness of disability is developing, for instance, we had a grant about gender equality and social inclusion in informal settlements to ensure that women and disabled people were included in terms of household and community decision making...the aspect of disability became very strong and that’s why we found out that we can partner with SLUDI to develop the UNDP programme [which has just been funded].”

(Man, stakeholder interviewee)

**Figure 3:**
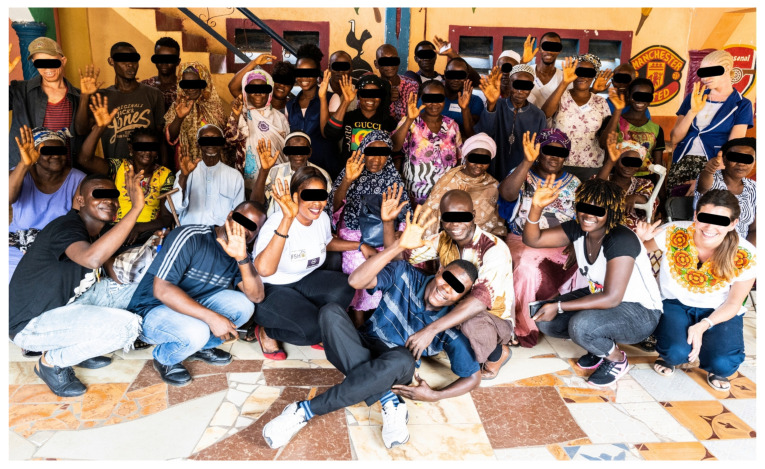
Dworzark Community, FEDURP data collectors and UCL research team after meeting together to talk about AT and disability issues the Community Center, November 2019. Credit: Angus Stewart.

Participants too, grew in in confidence to overcome stigma through their collective interactions at the weekly AT2030 meetings. The image below (Figure 3) was taken by a professional photographer, with active consent and with the intention of being shared in order to promote the visibility of slum dwellers. One participant said:

“Before [AT2030] I was ashamed of my visual impairment, but since this project has started, I now have the courage to speak, express myself and move around the community.”

(Participant at Disability Celebration Day, TB)

Connecting to each other—even knowing where to find each other now—was noted as an important change. In response to a speaker talking about feeling less ashamed, one participant added:

“Me too, I was ashamed to walk in certain areas within the community, but now is better.”

(Woman, TB-01)

This was mentioned by stakeholders too:

“I think the challenge is that Disabled People are in slums, but it’s difficult to identify them. That’s why we are very much pleased with the AT project. This has been the first time we have been able to work with disabled people in a community. It has been very more difficult for us in our (previous) mobilisations…”

(Man, stakeholder participant)

The celebration events held for International Day of Persons with Disabilities (2019 and 2020) raised awareness and profile, hitting the front page of the Freetown papers in 2019. Participants valued this recognition, in 2019 one participant commented:

“This is the first time of celebrating this day especially at community level which has been a remarkable day and this has created the space to feel belonging and make new friends and knowing that disability is not inability [one of the event slogans].”

(Participant at Celebration Day event, Dworzark)

The celebrations were observed, by the researchers, to raise the positive association around disability and overturn the pejorative association. One participant summed it up well:

“This is our home and we can show how much we are capable of doing. Just because you are small you can still participate.”

(Woman, TB-03)

What we have established so far, is that civic participation was important to slum dwellers with disabilities, but that both a lack of AT (especially to overcome the inaccessible environment), and stigma often prevented this participation. So now we turn to look at the availability of AT to the participants.

### 4.4. Difficulty in Accessing Appropriate AT, Leading to Very Little Use

First we examine the results of the rATA survey.

#### 4.4.1. Prevalence of AT Need and Availability

##### rATA Survey

A high prevalence—20.6% (429 people)—of the respondents reported ‘some difficulty’ or more in seeing, hearing, walking, remembering or concentrating, self-caring, speaking or communicating with a further 4.3% (91 people) experiencing a lot of difficulty and 0.4% (9 people) reporting they could not do one or more of these things at all.

Dworzark reported a higher prevalence of ‘some difficulty or more’ than Thompson Bay (25.9% D–15.1% TB). The most significant difference was in seeing; almost three times as many residents in Dworzark had difficulty seeing than in Thompson Bay. One explanation could be that the Dworzark settlement was surveyed first (during the first weeks of September) in which there is a higher prevalence of eye infection due to rainy season and this could relate also to topography. However, this does not explain all of the data. In the domains of remembering or concentrating; self-caring; and speaking or communicating Dworzark settlement has double the number of positive reports than in Thompson Bay. These types of impairments are less visible and potentially less recognised but although the prevalence is low in the population overall (less than 4.3%- D or less than 0.4%—TB), such as difference remains a significant finding between settlements and more research is needed to understand this. Combining results from both settlements, the most common difficulties identified related to seeing/vision (10.5%); Mobility (10.1%); and remembering or concentrating (3.1%).

More than half (51%) of the difficulties overall were in seeing. Difficulty in seeing increases consistently with age, both in men and women, however, men over 70 years old showed the most difficulty. Overall, eight out of ten men of that age have ‘some difficulty’ seeing, or more (84.6%), while five out of 10 (50%) women have difficulty seeing in the age bracket.

Population need (for AT) as determined in the rATA was estimated based on that the total answering “some difficulty”, “a lot of difficulty” and “cannot at all” against one of the functional domains (seeing, hearing, etc.). AT coverage was calculated based on mapping need against products identified in the two settlements. This was significantly low; for people reporting “some difficulty”, just under one-tenth (9.8%; *n* = 33) had an AP, while about a third of individuals who experienced “a lot of difficulty” (35.4%; *n* = 29), and just over a fifth who reported that they “cannot do at all” (22.2%; *n* = 2) had access to an AP.

Variety was also low. The survey only found 6 types of APs from the WHO list of prioritized APs [1], which totals 26 unique devices.

The total APs found in the two communities all related to mobility and seeing, they were:

- 52 pairs of spectacles (representing 81% of total APs)
- 3 axillary or elbow crutches
- 1 manual wheelchair
- 1 rollator and walking frame
- 1 therapeutic footwear

Women tended to have less sophisticated and less varied AP. Women had spectacles, auxiliary/elbow crutches and walking aids. In the case of men, they also had manual wheelchairs and rollators/walking frames. Affordability is the main reported reason for not having AT. A very large proportion (80.5%) of the respondents that needed AT said they did not have it because they could not afford it. Other options included not being aware, not available, not suitable or due to stigma. The RATA (and the training of data collectors) showed that people were not aware of different products and what they could do. For example, 100% of blind people said that they need eye glasses—instead of a white cane.

Overall, 174 of people surveyed said that they needed an AP that they did not currently have, which represents 40.1% of the people that reported some difficulty or more in any one domain, and only 14.9% of the population that needed an AP had access to any product at all, not accounting for its quality.

##### First-Hand Accounts of Participants

When interviewed individually, participants concurred with this survey data and specifically mentioned access to AT often, with one hundred percent confirming that AT was too expensive and hard or impossible to obtain for instance:

“when we went to the hospital the price of my AT was very expensive.”

(Woman, D-03)

This was mentioned in all individual interviews and in all focus groups. Where AT was being used by these participants, it was universally of a very poor quality. It was common to have to purchase these poor-quality products through necessity, creating a cycle of costly repairs and potential harm from ill-fitting or inappropriate APs:

“My parents bought me a crutch…but was very expensive (150,000 leones, about £13)…and it has a snapped armrest…it costs about 30,000 Leones (£2.50) to replace the rubber feet….Without my crutches, I couldn’t go anywhere.”

(Young Female, TB-01)

Some of the products observed were of great age; a few had been provided when Humanity and Inclusion (HI) gave out equipment without cost, more than a decade ago. In the case of one young man in Dworzark he was walking with a prosthetic that caused considerable pain. He received his prosthetic from an NGO, probably HI:

“I got my leg in 2007, I think it came from France.”

(Man, D-05)

Many products used by participants in data B, were of such low quality as to render them unusable or harmful, including one older man with a wheelchair that hadn’t wheeled for many years. It had been found for him randomly some years before. He was not able to move independently:

“In the morning my son moves me from my bed to outside my home [shack structure] under the tree, sitting in the [broken, static] wheelchair. I am more comfortable sitting there because it has a lot of wind blowing and shade, so it helps me a lot. After 7:00 pm, my son takes me back into my bedroom.”

(Man, TB-4)

In contrast to the overall picture of lack of awareness of AT shown in the RATA, Data B indicated that some participants were aware of the AT that could be helpful to them and felt entitled to access it. This may be due to timing, as the RATA had already been carried out when the citizenship study took place, so participants had all been shown the WHO APL list [1]. But participants also reported that their awareness was also driven by initiatives they read about in newspapers or watched on TV or media indicating that AT was on the way. Despite public awareness raising about AT available, participants questioned whether this would result in people actually accessing AT and felt frustration, as is evident:

“We always hear on the news that there are things coming for us…but we only get the news….”

(Man, TB-08)

Participants were unhappy, and sometimes despairing, about not being provided with the AT they needed and felt they deserved:

“You hear these things like wheelchairs have arrived for the disabled (sic)’... but when you go to the office…they never say when they are going to distribute them.”

(Man TB-8)

In addition, a need for the implementation of free healthcare, rehabilitation and basic essential medicines to manage conditions was also recorded, to enable participation:

“You go to the hospital and you will have to buy everything...when the money finish they will discharge you whether you get better or not…. If you have the medicine but not the needle, they will not attend to you.”

(Woman, D-02)

Because AT was, on the whole, unavailable to those who needed it, often participants had to rely on help from others in the community. Being forced to ask for help fetching water or getting around the settlement compounded issues of lower status, or lower perceived value of persons with disabilities in the community because of having to rely on others. If this help wasn’t available, combined with the absence of AT, there were direct consequences:

“I have seen so many people that didn’t have a helper pass away.”

(Woman, D-03)

It was easier to elicit help in the absence of AT when the status of the person needing it was greater, e.g., they were a church elder or had contributed to the community in some way. The examples we found of this were older men. One such example is an older man:

“I used to be a farmer...and after the farming year I would come with some gifts for the community…now I am blind but… [the young people]... they read the chapter of the Bible for me and I interpret it for them.”

(Man, TB-03)

In lieu of a fully functioning AT access system, the informal provision of support within the community became necessary for essential activities. We also investigated the data on informal provision of AT.

#### 4.4.2. Reliance on Ad Hoc and Informal Provision

Data B showed that participants who did have AT often bought products themselves from informal shops or markets:

“The government is not helping us. In the past, at the place at Aberdeen (informal shop) the AT was not much expensive, but now it is very much expensive to buy the AT products.”

(Man, D-01)

The rATA (Data A) also found that the informal (unregulated) sector was the largest provider of AP with one-third (30.8%) of the respondents obtaining their AP—mostly spectacles—from the informal sector. This included secondhand shops, markets and street hawkers (from whom it is also usual to buy medicines). Government facilities were the second most reported source of products (27.7%), such as government hospitals. Respondents with some functional difficulties were most likely to get their products from the informal sector (45.5%, *n* = 15), whereas people with a lot of difficulty tended to obtain them from government facilities (37.9%, *n* = 11), which could be explained by the sophistication and price of the product (wheelchair versus spectacles for instance).

Information about where to purchase products from was often informally shared between users who might potentially need them, indicating the fragility of information and service provision, as well as the realities of actual product provision in the AT system:

“I only hear through rumour, from friends and others, some usually said where they bought their AP”

(Man, D-08)

Replacements and repair, in addition to purchase, were also often made through the informal sector to keep costs down, though it was still considered expensive:

It costs about 30,000 (£2.50) to replace the rubber feet [for the crutches] from the market.

(Woman, TB-01)

Data C provided further evidence of the importance of what made up the informal provision and noted NGOs, Organisations of Persons with Disabilities and religious organisations all played a key role in providing basic AT, which—although cheaper—potentially had a range of disadvantages in terms of quality and appropriateness of provision (according to the WHO 5P’s framework for AT [1].

#### 4.4.3. There Was a Lack of Information and Coordination at Provision at National Level

Our interviews with participants (Data B); the RATA (Data A); and Informal Markets Study (Data C) all indicated national provision of AT did not have the coverage required to meet need. We went to the summary data from the (unpublished) Country Capacity Assessment to cross-reference, however no nationally representative data existed. Rather, the lack of national procurement, service provision or need data was a data point in itself. Stakeholders surveyed for the Capacity Assessment by CHAI, concurred in their perception of a significant gap between demand and supply of AT in the country.

Estimating AT need was acknowledged by those surveyed to be further complicated as the official estimates of persons with disabilities in SL are largely limited to the (contested) Population and Household Census (2015) [42] which estimates there are only 93,129 disabled people in the country (with an additional 9% of the population (of 7,076,119) over 50 [ibid].. There was also no comprehensive register of provision across departments, as responsibility for AT provision is shared between the Ministry of Health and Sanitation (AT services, AT standards, training and rehab services); the Ministry of Social Welfare (social protection), Gender and Children’s Affairs (disability, social protection); the Ministry of Education (AT for learning); and the National Commission for Persons with Disabilities (scrutiny and policy advice). GoSL estimated that the National Rehabilitation Center provides 500 wheelchairs per year and highlighted local NGO’s—like Mobility Salone based in Bo—who develop locally produced products, including some specialist products, but there is no comprehensive register of provision, cost or units distributed.

The Capacity Assessment resulted in an Action Plan, which the Government of SL used to identify their AT priorities, given what they learned through the assessment. The unpublished assessment provides a succinct summary of the key areas for prioritized for potential intervention and underlines the need for more coordination to improve AT provision. The Government of SL has invited stakeholders to join a National Disability, Assistive Technology and Rehabilitation Technical Working Group to help oversee a new ‘National Assistive Technology Programme’ which is currently under development to streamline fragmented services and agree a budget line for AT for the first time. These will seek to address the limited supply of personnel (with only 17 physiotherapists in the country, for instance); service provision; procurement proliferation; and coordination of activity which are all highlighted as priorities. The upgrading of the National Rehab Center; the development of a SL Priority Assistive Products list (APL); and the use of an IT based platform are all planned to improve supply chain management and enhance human resources (skills) in both the formal and informal market. These priorities align well with the evidence of need we gathered through other elements of our study, although we cannot corroborate prioritization or ranking from our data.

## 5. Discussion

### 5.1. Research Discussion 

This study set out to understand the meaningful activities of citizenship valued by the group, how AT mediated participation, and access to AT. The role of AT in mediating participation in the activities of citizenship for the poorest persons with disabilities was investigated through the use of multiple data sets. In using such an approach, it was possible to interrogate both the experience of persons with disabilities who are resident in informal settlements, in the context of the systems which regulate AT provision in SL. Next, we discuss our findings in context.

Taking all of the results into account, our data suggest that a lack of access to AT was a significant factor in limiting the participation of slum-dwellers who are persons with disabilities in both formal and informal citizenship activities. Further, we find that these citizenship activities were valued for their potential in forging progressive change. In particular, we found the lack of AT, together with attitudinal barriers (stigma), as limiting of collective participation which the participants in our study wished to pursue. Similar to others, we found AT to be vital for participation for persons with disabilities, but our study makes an additional contribution by highlighting the need for AT for citizenship participation.

Access to AT was exceptionally low, and of poor quality, and we detected no evidence of a well-functioning system that could meet demand nor structured information which could map provision. This is not unusual in low-resource settings, and the Government of SL rightly suggests addressing this, along with building capacity and a budget line, as first principle priorities. Need figures are complicated by official data on disability which is extremely low by international standards, and our RATA data confirms a higher prevalence of need that existing data would imply.

Though the legal framework does largely allow for equal rights and formal citizenship participation for persons with disabilities, the moments for this participation—where opportunity touches the lives of slum dwellers with impairments—were extremely limited. The operational ‘weakness’ of the resource-poor SL state from the perspective of those whose lives are lived on the periphery (in informality) is no doubt contextually relevant. Because of this, necessarily our scope of investigation also looked at participation in informal citizenship practices. The informal governance arrangements of urban poor people were universally respected and ultimately considered a pre-requisite for recourse to a better future by the slum-dwellers with disabilities we worked with; both in terms of community provisioning of solution and help now, and collective action to drive change in the future.

Though much previous research has highlighted the need for persons with disabilities to be able to participate in issues that affect their everyday lives themselves this work rarely considers the role of AT in that process, in this context. This study offers a contribution towards opening that debate. The engagement of persons with disabilities within the movements and organisations of the urban poor remains unfortunately infrequent, and is largely unexplored by research. Further work might seek to unpack the meaning of citizenship conceptually and in more depth for this group and we are minded here to make reference to the emerging body of work on social and spatial disability justice in the context of the global south; building on Critical Disability Studies [53] Crip Theory [54] and new models of disability-inclusive development. Particularly, we see value in considering the role of AT in enabling the participation of persons with disabilities in pursuing ‘the things they value’ [14] within a Capabilities Model of Disability Inclusive Development [55] which will be important if one understands the “distribution of justice as a fundamental, participatory, and deliberative process wherein social values are developed and implemented by the people most affected (ibid., p25).

In addition to AT, our data also revealed the operation of other factors which limited participation, which we summarize as ‘stigma’ in this paper. In reality, stigma comprises a complexity of interaction between cultural assumptions; the perceived value of persons with disabilities; and nature of disability identity, in context [56,57]; nuances we picked up in the operation of stigma include gender and age disparities, and status in the community which appeared to mitigate some aspects of mistreatment. The findings of this work contribute to an increasing number of other studies of stigma, disability and informality (albeit without the AT ‘lens’)—particularly relevant [56] found stigma to significantly mediate the association between disability and higher levels of depression and low self-esteem in a similar setting. But this paper does not afford space to explore the construction of stigma to any great extent and further work is needed.

Borrowing from Fraser [58] one might summarize that issues of misrecognition (of identity), maldistribution (of access to resources, including AT), and the resulting mis-representation (or lack of representation in key citizenship activities) were incredibly present for slum dwellers with disabilities. This resulted in a rather literal ’dis-parity of participation’ for slum dwellers with disabilities, functioning as an operational model of injustice. The interventions necessary to dismantle this construction and recreate a disability justice model relevant for this context, deserves much further analysis.

Our results confirm that quality AT is both largely non-existent and very necessary for this particular group and should be viewed within the social context of human interaction; confirming the findings of other studies in similar settings [7] that help and peer support often function in practice around, and in lieu of, appropriate AT. The results are pursuant to the importance of the current global push on access to AT and in fact its urgent acceleration in the current global context [59].

### 5.2. Limitations and Further Research 

While the sample size of Data B (individual slum dweller interviews) may be perceived as small, it is appropriate for a qualitative study on a focused case and is presented alongside Data A and C to triangulate findings. One limitation of this study was the inability to carry out a one-to-one interview with a potential participant with a hearing impairment, as an interpreter was not available in the country (a data point in itself). She did participate through a friend in the focus group discussions for AT2030, which were observed for this study, and through drawing and photography used in these workshops. We could not run the data for different impairment types in this study though anecdotally some were easier to manage without access to AT (for instance upper arm amputation was less limiting than lower leg amputation for some) and we know that some AT was much cheaper and more available (e.g., spectacles or crutches) than others (wheelchairs). This was due to the absence of AT and would be an interesting basis for a future study. We further highlight that some of the tools we used, notably the rATA, have a focus on AP, and therefore identifying evidence regarding systems and services around absent products is challenging with these tools. However, we sought to overcome this (global problem) by combining our datasets and painting a picture or reality as the experience of slum dwellers. During the collection of Data C, it was not possible to fully follow the COREQ Standard Reporting Guidelines for Qualitative Research [60] due to a lack of some data fields, given the data is owned by Government of Sierra Leone.

We accept that another limitation of this study is the inability of the whole research team to return to SL post the start of the COVID pandemic and test our themes, codes and results with participants, though the local SLURC and FEDURP team remained present in the communities and some authors (AD) participated in the later events under strict COVID restrictions. We would have preferred to be able to able to test the emerging themes and codes directly with the participants in Data B as a team.

Finally, it must be stated that ‘citizenship’ itself was not a well-understood term. In our test interviews we iterated many approaches, and ultimately—as set out in methods—we were required to amend with a qualifier which may have prompted a particular response, though the reporting of activities we define here as citizenship activities though, remained consistently communicated. The issue with citizenship is more than just translation—though it is true that in Krio (local language) the word was not understood well. Citizenship is a contested term with many meanings, attempting to analyze a complex phenomenon (the nexus between disability, poverty and AT provision) through a contested lens (‘citizenship’) adds further complexity. We might have considered a more direct approach—political participation or collective action, or disability justice—and it will be interesting to explore this in future work.

### 5.3. Reflections Offered to Policy and Practice 

Our intention was for this study was to offer practical policy recommendations in order to support better outcomes for people living in similar situations in the future. As such, we offer the following reflections:

- A greater degree of nuance is needed in the global evidence base on AT to address the specific issues of persons with disabilities who live in informal settlements—often this is the poorest group who are most in need yet research about the lived experience of this group, and their voices, are infrequently heard.
- AT as an enabler of citizenship participation deserves a greater understanding to avoid a de-facto and reductive focus on economically productive activities such as learning and earning. This will require more investigation into the ‘ends’ AT users define as valued, for which AT provides the ‘means’.
- In building a plan for comprehensive AT access, the Government of Sierra Leone could be helpfully supported through international co-operation as per the CRPD; particular attention might be paid to the informal market, given its prevalence. Supporting informal market traders appropriately and continuing to engage with slum dwellers with disabilities and their communities as coordination and investment are prioritized, will be important.
- Further research into AT as a mediator of participation parity (understood here as a model of disability justice) could be considered. Citizenship should be tested further as the lens for this exploration.

## 6. Conclusions

This study has shown that though appropriate AT is almost entirely absent, it remains an important mediator of access to both formal and informal citizenship participation for persons with disabilities who live in informal settlements in Freetown, SL. Further, citizenship participation was valued as a means toward achieving a better future. AT was not the only mediator of participation; stigma and (negative) status issues around disability were also found to be present and significant. However, our study reveals some cause for optimism in terms of future participation for this group; reporting both a desire for collective participation among persons with disabilities who live in informal settlements and also increasing engagement between organisations of persons with disabilities and organisations of the urban poor. This study also reports increased political will and commitment from the Government of Sierra Leone to address access to AT. Taken together they offer the prospect of this group being better able to make their claims for justice in the future.

We conclude by suggesting that further work might consider AT for the purposes of citizenship participation in greater depth, especially for the poorest, as a necessary prerequisite for delivering expressed global commitments on disability justice and inclusion. Finally, policy-makers and donors might consider the specific role participation must play in disability justice, and question: with what technology, provided within which social support structures, is the recourse of disability justice for the poorest best enabled?

## Data Availability

The data presented in this study are available on request from the corresponding author. The data are not publicly available due to being part of ongoing research programme, and in the case of data C (capacity assessment) being owned by the Government of Sierra Leone.

## Appendix A. Interview Topic Guide—Data A

### Appendix A.1. Intro

- AT2030—consent and recording
- Reminder of research objectives

### Appendix A.2. Part 1: Initial Questions

- Name
- Age
- Gender
- Occupation/Education
- Where do you live and with whom?
- For how long have you lived in this community?
- What AT do you have and use (triangulate with RATA)?

### Appendix A.3. Part 2: Key Lines of Enquiry

Citizenship and the City/State:

(1) Can you describe what it means to you to be a Sierra Leonean—to be a citizen of SL?
  (a) What are the basic things a citizen of SL can expect?
  (b) What are the basic things a citizen of SL must do?
(2) What activities do you do, as citizen of Sierra Leone?
  (a) Do you need, or receive, any help in these activities e.g., from friends/family or assistive technology?
  (b) Are there any activities you would like to do but cannot?
(3) Do you think there are any differences for a disabled person in being a citizen of SL, compared to a non-disabled person?

Citizenship and the Settlement:

(4) What makes someone be considered as a community member in this settlement?
  (Prompts: norms, meetings, development projects, community revenue contribution—is it more than just residency?)
  (a) If committees/meetings—Are you involved in any community meetings? Do you attend? Does anyone ever raise disability issues?
  (b) Are you a member of any other groups in the community e.g., church/mosque/social?
(5) Are there any differences in the the things a disabled person will be required to do to be considered a community member?
(6) Are there any ways that disabled people are able to participate with other disabled people in the settlement? (e.g., groups for disabled people, meetings or informal activities)
  (a) If no…do you think its would be god to have this type of meeting? Or not necessary?
  (b) Why?
(7) If someone wanted to raise issues of concern to disabled people in the settlement how would they do that?
(8) If you had the opportunity to raise issues that are important to disabled people, here what are the top issues you would raise with the community/community leaders (or other people identified in 7)?

Participation and voice

(9) Can you tell me about whether /how things have changed in terms of participation for disabled people in the community throughout the time you have lived here?
(10) Where is it that you feel people listen to you and your voice is heard? (e.g., home, school, community, social media?)
(11) Where do you feel most able to be yourself? (translation: to be ‘comfortable’ and ‘feel fine’)

### Appendix A.4. Part 3: End Questions

- Is there anything else you’d like to tell me?
- Is there anyone else you think I should talk to?
- Is there anything else you’d like to ask about the research?

## Appendix B. Data Ca CCA Stakeholder Survey List

1. Directorate of NCDs and Mental Health, MOHS
2. National Assistive Technology Programme, MOHS
3. Ministry of Social Welfare Gender and Children’s Affairs (MSWGCA)
4. National Commission for Persons with Disability
5. Sierra Leone Union on Disability Issues
6. POPDA Makeni
7. WESOFORD Kambia
8. Mobility Sierra Leone Bo
9. Handicap International
10. SLEDT
11. 34 Military Hospital
12. MoHS/Sierra Leone Physiotherapy Association (SLPA)
13. MoHS/Sierra Leone Orthotic & Prosthetic Association (SLOPA)
14. MoHS/NRC
15. MoHS Connaught
16. MoHS BO Government Hospital
17. Koidu Regional Rehabilitation Center Koidu Govt Hospital
18. Emergency Hospital
19. MoHS BO Government Hospital
20. World Health Organisation
21. Abdul Miracle Disabled Children’s Foundation
22. Sierra Leone Association of the Deaf
23. Human Rights Commission Sierra Leone
24. Ministry of Education
25. Freetown Chercher Home
26. Disability Awareness Action Group
27. Amputee/War Wounded
28. One Family People Org. Freetown
29. Kings SL Partnership
30. Enable the Children
31. Latter Day Saint
32. NEHP/MoHS
33. Life Matters Disabled Foundation
34. Sierra Leone Urban Research Center

## Appendix C

**Table A1:** Final Coding Framework.

1	2	3	4	5	6 (Added)
Status of DP in Community	Shame/Sense of Belonging	Expectations of Citizenship	Active Citizenship Practices	(Lack of) Collective Participation on Disability or by DP	AT Provision and Need
Begging	Asking for help	Aspirations/hopes	Community collective participation	Disability and citizenship in reality	AT need
Helping to gain respect	Discrimination/mockery	AT provision (moved to 6)	membership of the community	ODP and representation	AT provision
Status in the community (combining age, gender and owner/tenant)	(lack of) Recognition	Broken promises	How to make change	Political Participation	Informal provision
	Self-perception mindset	Common community issues	Reliance on community action		National capacity
	Sense of belonging	Expectations	Tackling disability issues in the city		AT ‘for what’?
	Shame	Media/culture			
